# Value-Based Purchasing Financial Outcomes Among Minority-Serving Skilled Nursing Facilities

**DOI:** 10.1093/geroni/igaa057.146

**Published:** 2020-12-16

**Authors:** Jennifer Gaudet Hefele, Hari Sharma, Bryant Conkling, Lili Xu, Xiao (Joyce) Wang

**Affiliations:** 1 Booz Allen Hamilton, Hollis, New Hampshire, United States; 2 The University of Iowa, Iowa City, Iowa, United States; 3 Booz Allen Hamilton, Bethesda, Maryland, United States; 4 University of Iowa, University City, Iowa, United States; 5 University of Massachusetts, Boston, Massachusetts, United States

## Abstract

Skilled Nursing Facility (SNF) Value-Based Purchasing (VBP) ties post-acute payments to readmissions performance. Minority-serving SNFs tend to be poorly resourced and understanding the financial implications of SNF-VBP is important. Our study examined VBP payments and penalties among minority-serving SNFs. We conducted cross-sectional analysis using public data sources. We defined minority-serving as SNFs with >50% of residents who are African American (AA)/Black (n=764) or with >50% of residents who are Hispanic/Latino (n=164). Majority-White SNFs (>50% residents who are White) were the reference group (n=11,002). Outcomes examined were: being in top 20% of performance rankings; receiving a bonus; receiving a bonus that was above the median bonus dollar amount; being in bottom 20% of performance rankings; receiving a penalty; receiving a penalty that was below the median penalty dollar amount. Logistic models estimated the likelihood of experiencing each performance outcome for AA/Black-serving and Hispanic/Latino-serving SNFs in reference to majority-White SNFs. Results show that minority-serving SNFs not only perform worse but also experience greater negative financial impacts. Among those penalized, Hispanic/Latino-serving SNFs had the largest average penalty amounts: $32,575 compared to approximately $26,000 for both AA/Black- and White-serving SNFs. Hispanic/Latino-serving SNFs had 1.69 times the odds of receiving larger than median penalties and AA/Black-serving SNFs had 27% lower odds of receiving higher than median bonus payments. Average penalties approximate the average salary for a certified nursing assistant, the primary direct care worker in this setting. Results from this study raise concerns over long-term impacts. Alternative approaches to encouraging quality should be considered.

